# Drug-resistant strains of *Mycobacterium tuberculosis*: cell envelope profiles and interactions with the host

**DOI:** 10.3389/fcimb.2023.1274175

**Published:** 2023-10-27

**Authors:** Alyssa Schami, M. Nurul Islam, John T. Belisle, Jordi B. Torrelles

**Affiliations:** ^1^ Population Health Program, Texas Biomedical Research Institute, San Antonio, TX, United States; ^2^ Integrated Biomedical Sciences Program, University of Texas Health Science Center at San Antonio, San Antonio, TX, United States; ^3^ Mycobacteria Research Laboratories, Department of Microbiology, Immunology and Pathology, Colorado State University, Fort Collins, CO, United States; ^4^ International Center for the Advancement of Research & Education, International Center for the Advancement of Research & Education, Texas Biomedical Research Institute, San Antonio, TX, United States

**Keywords:** Mycobacterium tuberculosis, tuberculosis, drug resistance, cell envelope lipids, M.tb-host interactions

## Abstract

In the past few decades, drug-resistant (DR) strains of *Mycobacterium tuberculosis* (*M.tb*), the causative agent of tuberculosis (TB), have become increasingly prevalent and pose a threat to worldwide public health. These strains range from multi (MDR) to extensively (XDR) drug-resistant, making them very difficult to treat. Further, the current and future impact of the Coronavirus Disease 2019 (COVID-19) pandemic on the development of DR-TB is still unknown. Although exhaustive studies have been conducted depicting the uniqueness of the *M.tb* cell envelope, little is known about how its composition changes in relation to drug resistance acquisition. This knowledge is critical to understanding the capacity of DR-*M.tb* strains to resist anti-TB drugs, and to inform us on the future design of anti-TB drugs to combat these difficult-to-treat strains. In this review, we discuss the complexities of the *M.tb* cell envelope along with recent studies investigating how *M.tb* structurally and biochemically changes in relation to drug resistance. Further, we will describe what is currently known about the influence of *M.tb* drug resistance on infection outcomes, focusing on its impact on fitness, persister-bacteria, and subclinical TB.

## 1 Introduction

Tuberculosis (TB), the disease caused by the pathogen *Mycobacterium tuberculosis* (*M.tb*), is currently estimated to cause ~1.6 million deaths annually worldwide and is considered a leading cause of death due to a single infectious organism (WHO, 2022a). Recently, the World Health Organization (WHO) updated their definitions of drug-resistant (DR)-TB along with the newest approved treatment regimens being implemented. As defined, multi drug-resistant (MDR)-TB is caused by a *M.tb* strain with resistance to isoniazid (INH, H) and rifampicin (RIF, R). Pre-extensively drug-resistant (pre-XDR)-TB is caused by a *M.tb* strain resistant to RIF (may also be resistant to INH) and to at least one fluoroquinolone [levofloxacin (LEV) or moxifloxacin (MFX, M)]. Lastly, *M.tb* strains that cause extensively drug-resistant (XDR)-TB are resistant to RIF (may also be resistant to INH), at least one fluoroquinolone (LEV or MFX), and one “Group A” drug [bedaquiline (BDQ, B) or linezolid (LZD, L)] (**Table 1**) (WHO, 2022c). From this point on, the one letter drug abbreviation will only be used when referring to a combined treatment.

**Table 1:** Updated drug resistance categories based on the new WHO definitions (WHO, 2022c).

Drug Resistance Category	Resistant to:			
	Rifampicin	Isoniazid	Levofloxacin or Moxifloxacin*	Bedaquiline or Linezolid**
**Rifampicin resistant (RR)**	**+**	**-**	**-**	**-**
**Multi drug-resistant (MDR)**	**+**	**+**	**-**	**-**
**Pre-extensively drug-resistant (Pre-XDR)**	**+**	Possibly	**+**	**-**
**Extensively drug-resistant (XDR)**	**+**	Possibly	**+**	**+**

*At least one fluoroquinolone drug.

**At least one “Group A” drug. "+" signifies resistance, "-" signifies susceptibility.

Standard anti-TB treatment regimens for both drug-susceptible (DS)- and DR-TB were also recently updated by the WHO in May 2022. Previously, treatment for DS-TB included a 2-month intensive phase of treatment with INH, RIF, pyrazinamide (PZA, Z) and ethambutol (EMB, E), followed by a 4-month continuation phase with just INH and RIF (2HRZE/4HR) (WHO, 2022b). The new WHO recommendation for DS-TB treatment also includes implementation of a newer, 4-month daily treatment regimen consisting of rifapentine (RPT), INH, PZA, and MFX that is non-inferior to the 6-month DS-TB treatment regimens (**Table 2**) (Carr et al., 2022, WHO, 2022b). The WHO updated recommendations for DR-TB treatment includes three separate treatment options for diagnosed patients. Choice of DR-TB treatment regimen is based on factors such as the *M.tb* drug-resistance profile, prior exposure to anti-TB drugs, and the extent of pulmonary TB disease among others (WHO, 2022c). Of the three possible treatment regimens, the newest and preferred DR-TB regimen for RIF-resistant (RR)/MDR-TB cases consists of BDQ, pretomanid (Pa), LZD, and MFX (BPaL+ M) as an all-oral regimen that lasts 6 months. For pre-XDR-TB cases, the same treatment regimen is recommended without MFX (Nyang'wa et al., 2022, WHO, 2022c). An additional recommended treatment for MDR/RR-TB is a 9-month all-oral regimen consisting of BDQ for 4-6 months in combination with a fluoroquinolone, ethionamide (ETH), EMB, INH (high dose), PZA, and clofazimine (CFZ), and treatment with a fluoroquinolone, CFZ, EMB, and PZA for the remaining 5 months (WHO, 2022c). Finally, a longer individualized treatment regimen is recommended for patients with DR-TB that have not had desirable outcomes with other regimens, are intolerant to specific components of the other regimens, or have XDR-TB (WHO, 2022c). These individualized regimens last at least 18 months and have poor adherence to due to the long duration and the increased likelihood of adverse side effects; thus, one of the other two regimens is preferred if possible.

**Table 2:** Updated treatment regimens for each disease classification based on the new WHO recommendations (Carr et al., 2022; Nyang'wa et al., 2022, WHO, 2022b, WHO, 2022c).

Disease Classification	Treatment Regimen	Length of Treatment	All-Oral	Age Recommendation
**DS-TB**	2HRZE/4HR	2 months of HRZE; 4 months of HR (6 months total)	Yes	All ages
	2HPMZ/2HPM	2 months of HPMZ; 2 months of HPM (4 months total)	Yes	People ≥ 12 years old
**MDR/RR-TB**	BPaLM*	6 months	Yes	People ≥ 14 years old
	9-month all-oral	9 months	Yes	All ages
**Pre-XDR-TB**	BPaL	6 months	Yes	People ≥ 14 years old
MDR/RR-TB,** pre-XDR-TB,** or XDR-TB	Longer individualized	18 months	No	All ages

*Preferred method of treatment for MDR/RR-TB.

**Longer individualized treatment regimen only suggested if other treatment regimens cannot be used.

H, Isoniazid; R, Rifampicin; Z, Pyrazinamide; E, Ethambutol; M, Moxifloxacin; P, Rifapentine; B, Bedaquiline; Pa, Pretomanid; L, Linezolid.

Many anti-TB drugs target *M.tb* cell envelope biogenesis (**Table 3**). However, the mechanisms used by *M.tb* to regulate its cell envelope composition and the outcomes in relation to drug resistance (and, consequently, how this regulation influences TB pathogenesis) are poorly understood. Various studies have examined the complex cell envelope of *M.tb* and show that there are differences in lipid composition that are strain specific (Torrelles et al., 2004; Torrelles et al., 2008; Torrelles et al., 2011; Howard et al., 2018). Thus, in order to advance the TB field, it is required that we increase our understanding of similarities and/or differences in the cell envelope composition of DR-*M.tb* strains and how these changes influence bacteria-host cell interactions and infection outcomes. In this review, we will present what is currently known about how the *M.tb* cell envelope changes in relation to drug resistance, examine the influence that drug resistance has on *M.tb* infection outcomes, and address some of the gaps in knowledge that remain to be elucidated.

**Table 3:** Mechanisms of action of Anti-TB Drugs.

Drug Target	Drug	Mechanism(s) of Action	Treatment Regimen*	References
**DNA/RNA/Protein Synthesis**	RIF	Inhibits *M.tb* DNA-dependent RNA polymerase to suppress RNA synthesis	DS-TB	Campbell et al., 2001; Tupin et al., 2010
	RPT	Inhibits *M.tb* DNA-dependent RNA polymerase to suppress RNA synthesis (similar to RIF); Longer half-life, lower MIC, and higher protein binding than RIF	DS-TB	Alfarisi et al., 2017; Zheng et al., 2017
	MOX	Inhibits *M.tb* topoisomerase II (DNA gyrase)	DS-TB or MDR/RR-TB	Naidoo et al., 2017
	LNZ	Prevents the formation of functional bacterial 70S ribosomal initiation complex to inhibit bacterial protein translation	MDR/RR-TB or Pre-XDR-TB	Diekema and Jones, 2000
**Cell Envelope Permeability**	INH	Competitively inhibits InhA through the covalent formation of the INH-NAD adduct to prevent MAc biosynthesis	DS-TB	Wilson et al., 1995; Chen et al., 2002; Broussy et al., 2003; Schroeder et al., 2005; Vilchèze and Jacobs, 2007
	PZA	Pyrazinoic acid (active form of PZA) inhibits fatty acid synthesis by interfering with FAS I, blocks ATP production, and inhibits *M.tb* protein synthesis	DS-TB	Ngo et al., 2007; Zimhony et al., 2007
	EMB	Inhibits *M.tb* arabinosyltransferases to interfere with the biosynthesis of arabinose-containing cell envelope components and prevent bacterial division	DS-TB	Escuyer et al., 2001; Zhang et al., 2003; Amin et al., 2008; Goude et al., 2009; Zhang et al., 2020; Lee and Nguyen, 2023
	Pa	Inhibits MAc biosynthesis in aerobic conditions; Causes respiratory poisoning of *M.tb* via the nitrosylating effects of nitroimidazole, which release reactive nitrogen species under anaerobic conditions	MDR/RR-TB or Pre-XDR-TB	Singh et al., 2008; Thompson et al., 2017
	ETH	Competitively inhibits InhA (similar to INH) through the covalent formation of the ETH-NAD adduct to prevent mycolic acid biosynthesis	MDR/RR-TB	Banerjee et al., 1994
	DLM	Inhibits methoxy- and keto-mycolic acid synthesis via the F420 coenzyme system and generates nitrous oxide via the nitrosylating effects of nitroimidazole	MDR/RR-TB	Singh et al., 2008; Khoshnood et al., 2021
**Energy Production**	BDQ	Inhibits the activity of mycobacterial ATP synthase	MDR/RR-TB or Pre-XDR-TB	Sarathy et al., 2019
	CFZ	Interferes with K^+^ uptake and ATP production by interacting with *M.tb* membrane phospholipids to destabilize the membrane	MDR/RR-TB	Cholo et al., 2012

*Longer treatment regimens for DR-TB may use an individualized combination of drugs listed based on previous outcomes with specific drugs, intolerance, or the diagnosis of XDR-TB.

RIF, Rifampicin; RPT, Rifapentine; MFX, Moxifloxacin; LZD, Linezolid; INH, Isoniazid; PZA, Pyrazinamide; EMB, Ethambutol; Pa, Pretomanid; DLM, Delamanid; ETH, Ethionamide; BDQ, Bedaquiline; CFZ, Clofazimine.

## 2 The *M.tb* cell envelope

**Figure 1:**
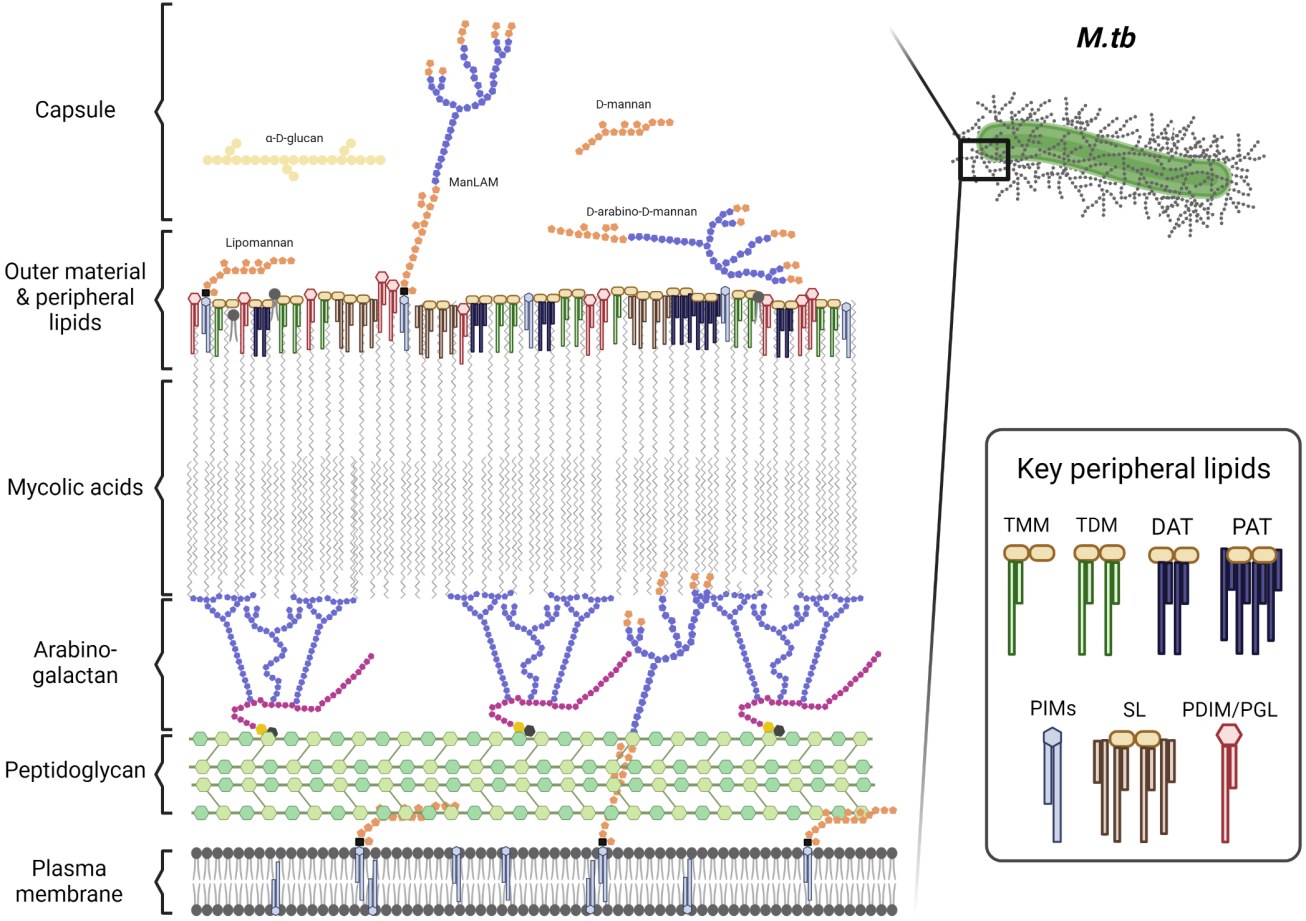
The *M.tb* cell envelope. Adapted from Mary Jackson (Jackson, 2014). TMM, trehalose monomycolate; TDM, trehalose dimycolate; DAT, diacyl-trehalose; PAT, pentaacyl-trehalose; PIMs, phosphatidyl-*myo*-inositol mannosides; SL, sulfolipids; PDIM, phthiocerol dimycocerosates; and PGL, phenolic glycolipids. Cell envelope components size are not depicted at a proportional scale. Figure created in BioRender.

The *M.tb* cell envelope is a thick and complex structure that provides a unique barrier of protection to the bacterium in various environments (Garcia-Vilanova et al., 2019). It is comprised of multiple layers that each contribute to its complex composition (detailed in **Figure 1**, adapted from (Jackson, 2014). The innermost layer is a plasma membrane similar to that of many other bacterial species (Jackson, 2014). Outside of the plasma membrane, a layer of peptidoglycan (PG) is covalently attached to the arabinogalactan (AG) polysaccharide. The non-reducing ends of AG are esterified to the long-chain (C_60_-C_90_) mycolic acids (MAcs) that are unique in nature to *Mycobacterium spp* and complete the ‘cell wall core’ of the cell envelope (Jackson, 2014; Garcia-Vilanova et al., 2019). Non-covalently bound lipoglycans, glycolipids, and lipids span the MAcs and create a peripheral lipid layer. On the top of this peripheral lipid layer the outer material ‘*capsule*’ is composed of α-glucan, arabinomannan, mannan, and several proteins among others. The peripheral lipid layer includes key lipid classes known to influence infection outcomes such as phthiocerol dimycoserosates (PDIMs), phenolic glycolipids (PGLs, present in some *M.tb* strains), trehalose monomycolate (TMM) and dimycolate (TDM), diacyl/triacyl/pentaacyl-trehaloses (DAT/TAT/PAT), sulfolipids (SL-1), and phosphatidyl-*myo*-inositol mannosides (PIMs) and their associated lipoglycans such as lipomannan (LM) and mannose-capped lipoarabinomannan (ManLAM) (**Figure 1**) (Daffe and Etienne, 1999; Constant et al., 2002; Reed et al., 2004; Tsenova et al., 2005; Reed et al., 2007; Sinsimer et al., 2008; Jackson, 2014; Garcia-Vilanova et al., 2019). The importance of the peripheral lipid layer is underscored by the fact that it accounts for 40% of the *M.tb* cell envelope composition (Garcia-Vilanova et al., 2019).

The *M.tb* cell envelope is an evolving and adaptive component of the bacterium and is critical to its protection against adverse environmental conditions during infection (Garcia-Vilanova et al., 2019). Many anti-TB drugs such as INH and EMB target cell envelope biogenesis to break down this barrier and weaken the bacterium. Anti-TB drugs targeting the cell envelope can be bactericidal, allowing other drugs to go through. Importantly, many mutations that confer resistance to anti-TB drugs arise within these cell envelope biogenesis pathways, making the generation of new anti-TB drugs critical to combating the recent increase in DR-TB (Batt et al., 2020).

Overall, the *M.tb* cell envelope has many distinct layers that come together to give protection to *M.tb* in various environments and resistance to many different drugs (Jarlier and Nikaido, 1994; Jackson, 2014). These key lipids are described briefly below in the context of drug resistance, while a detailed description of the peripheral layer of the cell envelope is provided elsewhere (Garcia-Vilanova et al., 2019).

## 3 *M.tb* cell envelope targets for anti-TB drugs

Understanding the *M.tb* cell envelope is critical to recognizing the complexity of the anti-TB drug treatment regimens required to kill DS- and DR-*M.tb* (**Table 3**). For 2HRZE/4HR treatment regimen, INH is a prodrug and its activation is associated with reduction of *M.tb* ferric catalase-peroxidase KatG by hydrazine to form ferrous KatG. Then, ferrous KatG reacts with oxygen to form oxyferrous KatG to activate INH (Wang et al., 1998). Once activated, INH inhibits InhA, the *M.tb* enoyl reductase, by forming a covalent adduct with the NAD cofactor. This INH-NAD adduct acts as a slow, tight-binding competitive inhibitor of InhA to interfere with MAc biosynthesis (Wilson et al., 1995; Chen et al., 2002; Broussy et al., 2003; Schroeder et al., 2005; Vilchèze and Jacobs, 2007). RIF inhibits *M.tb* DNA-dependent RNA polymerase, leading to a suppression of RNA synthesis and bacterial death (Campbell et al., 2001; Tupin et al., 2010). PZA diffuses into active *M.tb* expressing the pyrazinamidase enzyme that converts PZA to the active form pyrazinoic acid, where it interferes with fatty acid synthase (FAS) I (Ngo et al., 2007; Zimhony et al., 2007). This hinders *M.tb*’s ability to synthesize new fatty acids required for bacterial growth and replication (Ngo et al., 2007). Accumulation of pyrazinoic acid also disrupts membrane potential by interfering with ATP production as well as binding to the ribosomal protein S1 (RpsA), inhibiting trans-translation and blocking *M.tb* proteins being synthesized with high fidelity. (Shi et al., 2011) EMB directly inhibits *M.tb* arabinosyltransferases (EmbA, EmbB, and EmbC), interfering with the biosynthesis of arabinose-containing *M.tb* cell envelope components such as AG and LAM, as well as preventing bacterial division (Escuyer et al., 2001; Zhang et al., 2003; Amin et al., 2008; Goude et al., 2009; Zhang et al., 2020; Lee and Nguyen, 2023). EMB is thought to have a major impact on the cell envelope structure and permeability, as the arabinan domain of AG sustains the MAc barrier (Goude et al., 2009). Thus, less or smaller/truncated arabinan domain in AG could decrease the binding sites for MAcs, leading to a thinner MAc barrier and the accumulation of free MAcs, TMM, and TDM (Takayama et al., 1979). Further, *M.tb* surface exposed LAM interacts with host cells; thus, EMB-derived truncated or reduced levels of LAM on the *M.tb* cell envelope may decrease *M.tb* interactions with phagocytes (Zhang et al., 2003; Torrelles et al., 2004; Kang et al., 2005; Torrelles et al., 2008). Overall, the 2HRZE/4HR treatment for DS-TB is designed to increase permeability by dismantling the *M.tb* cell envelope (INH, EMB, PZA), followed by a direct inhibition of RNA-polymerases (RIF) driving bacterial death.

In the case of the new WHO treatment regimen recommendation for DS-TB of 4-month daily treatment regimen including RPT, INH, PZA, and MFX, the substitution of RIF for RPT adds value in multiple ways. Although RPT also inhibits *M.tb* DNA-dependent RNA polymerase activity, it has lower minimum inhibitory concentration (MIC), a longer half-life, and higher protein binding affinity, which altogether results in shorter therapies with reduced cytotoxicity (Alfarisi et al., 2017; Zheng et al., 2017). Further, replacing EMB with MFX is motivated by the fact that MFX inhibits *M.tb* topoisomerase II (DNA gyrase) (Naidoo et al., 2017). This enzyme is essential, as DNA gyrase is involved in the replication, transcription and repair of *M.tb* DNA. Thus, adding RPT and MFX while keeping the bactericidal effects of INH and PZA on the *M.tb* cell envelope is intended to maximize the effects of RPT and MFX on the bacterium.

For DR-TB, the newest oral treatment regimen approved is BPaL + MFX (for MDR/RR-TB) or BPaL alone for pre-XDR TB. The overall effects of the combination of these drugs on the cell envelope is unknown. However, BDQ is a diarylquinoline that inhibits the activity of mycobacterial ATP synthase that is essential for the generation of energy in *M.tb* (Sarathy et al., 2019). Pretomanid (Pa) inhibits *M.tb* MAc biosynthesis and causes respiratory poisoning of *M.tb* through the nitrosylating effects of nitroimidazole, causing the release of reactive nitrogen species under anaerobic conditions (Singh et al., 2008; Thompson et al., 2017, 2019). Pa molecular mechanisms of action may also involve effects on *fasI* and *fasII* (involved in mycobacterial fatty acid synthesis), as well as *efpA* (encoding the efflux pump EfpA implicated in the efflux of INH), the *cyd* operon (encoding cytochrome *bd* oxidase involved in the mycobacterial electron transport chain), and the *iniBAC* operon (INH induced operon involved in mycobacterial cell envelope stress responses) (Manjunatha et al., 2009). Finally, LZD interferes with bacterial protein translation by preventing the formation of the functional bacterial 70S ribosomal initiation complex, which is essential for *M.tb* proliferation (Diekema and Jones, 2000). Thus, the BPaL treatment regimen alters the *M.tb* MAc layer (biosynthesis), ATP production, and bacterial proliferation. These effects, together with the effects of MFX on *M.tb* DNA replication, transcription, and repair, initially makes BPaL + MFX a suitable treatment regimen for MDR/RR-TB. In this regard, other drugs such as ETH, delamanid (DLM), CFZ, and high doses of INH could also be used for the treatment of MDR/RR-TB. Increasing INH critical concentration aids in maintaining disruption of MAc biosynthesis. Further, ETH, like PZA, is a nicotinic acid derivative related to INH and thus, it acts similarly to INH where the covalent ETH-NAD adduct formed can act as a slow, tight-binding competitive inhibitor of InhA (Banerjee et al., 1994). DLM similarly inhibits the synthesis of methoxy- and keto-MAcs by disrupting the mycobacteria coenzyme F420 system and generating nitrous oxide through the nitrosylating effects of its nitroimidazole metabolites (Singh et al., 2008; Khoshnood et al., 2021). In the case of CFZ, its activity appears to be membrane-directed, interacting with *M.tb* membrane phospholipids to generate antimicrobial lysophospholipids which destabilizes the membrane, interfering with K^+^ uptake and ultimately ATP production (Cholo et al., 2012). However, the use of CFZ could be a double-edged sword, as it is also linked to local inhibition of host memory and effector T cell responses.

In summary, all anti-TB drug treatment regimens are developed to target *M.tb* cell envelope permeability, energy production, and DNA/RNA/protein synthesis (**Table 3**). The use of a combination of drugs in treatment regimens increases the permeability of the *M.tb* cell envelope, while simultaneously targeting key processes for survival. These are also necessary to prevent the emergence of drug resistance and shorten treatments. Treatment length and drug cytotoxicity drives drug misuse and provides *M.tb* with an ideal scenario to evolve mechanisms of drug resistance, potentially changing its cell envelope properties to its own benefit.

## 4 Specific *M.tb* cell envelope components and their relationship with drug resistance

The *M.tb* cell envelope has evolved over time to increase *M.tb* hydrophobicity, transmissibility, and pathogenesis during infection (Jankute et al., 2017). Indeed, some reports suggest that modern *M.tb* strains evolved from *M. canetti*, increasing their hydrophobicity and ability to be transmitted by increasing the amount of nonpolar lipids in comparison to polar lipids on the cell envelope (Fabre et al., 2004; Jankute et al., 2017; Briquet et al., 2019). However, while studies have examined the evolution of the *M.tb* cell envelope over time, there is still a large gap in knowledge related to how the *M.tb* cell envelope changes in relation to drug resistance. Here we summarize what is known about DS *vs*. DR-*M.tb* cell envelope changes and how they influence *M.tb*-host interactions.

### 4.1 Cell envelope thickness

As aforementioned, the *M.tb* cell envelope is a critical barrier of protection for the bacterium. It has also been shown to vary drastically between strains (Torrelles et al., 2004; Torrelles et al., 2008; Torrelles et al., 2011); therefore, it is logical to question whether such variation extends to *M.tb* strains in relation to drug resistance patterns. Some groups have examined the morphological similarities and differences between *M.tb* clinical isolates that range in drug resistance and found significant changes in morphology across strains (Velayati et al., 2018). Initial studies using transmission electron microscopy (TEM) showed significant increases in cell envelope thickness of both MDR- and XDR-*M.tb* strains compared to DS strains (Velayati et al., 2009a). Notably, MDR-*M.tb* strains are thicker in the electron-transparent and electron-opaque outer layer, while XDR-*M.tb* strains showed a denser peptidoglycan layer (Velayati et al., 2009a). In a subsequent study, the same group examined what they denote as totally drug-resistant (TDR)-*M.tb* strains using TEM, showing that these strains produce a thicker cell envelope and ‘*round/oval shaped*’ bacilli compared to both DS- and MDR-*M.tb* strains’ *rod shape* (Velayati et al., 2009b). Atomic force microscopy (AFM) produced similar findings of increased cell envelope thickness and surface roughness of *M.tb* strains denoted as extremely drug-resistant (XXDR) when compared to DS-*M.tb* strains (Velayati et al., 2010). Both TEM and AFM studies were able to reproduce XDR- and XXDR-*M.tb* strains’ round bacilli as well as a fourth “*adaptive*” type of cell division not found in DS- or MDR-*M.tb* clinical isolates (Farnia et al., 2010; Velayati et al., 2010). These studies suggest that in contrast to the three other types of cell division found in all *M.tb* clinical isolates (symmetrical, asymmetrical, and branching), this fourth adaptive type resulted in the ‘*round shaped’* bacilli and may be due to the bacteria’s efforts to protect itself and survive within a drug-driven hostile environment (Farnia et al., 2010). Indeed, these changes in *M.tb* cell envelope structure and shape were later associated with dormancy, where in the first 18 months of using an anaerobic culture to model latency, *M.tb* showed a thickened cell envelope and formation of the ‘*round shaped spore-like*’ bacilli (Velayati et al., 2011). This suggests that the shape, size, and thickness of the *M.tb* cell envelope may also be related to the amount of time that the bacteria remain latent during infection, and not solely in relation to drug resistance (Velayati et al., 2011). Importantly, many of these studies predate the most recent WHO definitions of TB drug resistance, so *M.tb* strains previously denoted as XXDR or TDR are now categorized as pre-XDR or XDR under the new definitions (WHO, 2022c). This is critical to note for future studies that will examine the structural characteristics of DR-*M.tb* strains.

### 4.2 Cell envelope lipid composition

While the modern *M.tb* strains cell envelope has increased hydrophobicity compared to more ancient mycobacterial strains such as *M. canetti* and *M. kansasii* (Jankute et al., 2017), not much is known about whether a further increase in hydrophobic lipids on the *M.tb* cell envelope in relation to drug resistance occurs. A few studies examined the prevalence of glycolipids and lipoglycans that range in polarity and have found variations between *M.tb* strains that are DS *vs.* DR. For instance, one study showed that LAM structural variations (*e.g.* highly succinylated and truncated arabinan domain with lesser mannose caps) in an EMB-resistant strain of *M.tb* was responsible for influencing host responses (Torrelles et al., 2004). Further, another group compared MDR- *vs.* DS-*M.tb* strains and found that there were critical differences between three major classes of lipids, namely on fatty acyls (e.g, MAc), glycerolipids (e.g., TAGs), and glycerophospholipids (e.g., PIMs) (Rahul Pal et al., 2015; Pal et al., 2017). A separate study looking at the lipid composition of INH-resistant *M.tb* clinical isolates found that these had notably lower α-MAc, PDIMs, TMM, and PIMs when compared to laboratory DS-*M.tb* strains (Nieto et al., 2018). However, changes to cell wall composition is not specific to the cell wall targeting anti-*M.tb* drugs, as one group revealed an increase in expression of genes responsible for PDIM biosynthesis and alteration in PDIM structures in strains with *rpoB* mutations resulting in RIF resistance (Bisson et al., 2012). While these studies suggest that the lipid composition of DR-*M.tb* strains is altered, a consensus of how the cell envelope of DR-*M.tb* strains change in comparison to DS-*M.tb* strains remains to be established. Below we will discuss MAcs along with key subsets of *M.tb* peripheral lipids and what is known about each lipid subset in relation to drug resistance.

#### 4.2.1 Mycolic acids

MAcs are long-chain (C_60-90_) fatty acids that are covalently bonded via esterification to the non-reducing ends of AG to complete the *M.tb* cell wall ‘core’ (Jackson, 2014; Garcia-Vilanova et al., 2019). They are a key component of the mycobacterial membrane and critically contribute to the hydrophobicity of the cell envelope which protects the *M.tb* bacterium during infection and from anti-TB drugs. Studies comparing the cell wall structure of *in vivo* and *in vitro* derived *M.tb* have shown that *in vivo* grown cells have a more condensed AG-PG structure, but with increased esterification by mycolic acids. *M.tb* produces three subclasses of MAcs (α-, methoxy-, and keto-MAcs) (Nataraj et al., 2015). In addition to being covalently bound, MAcs can be found free and esterified to peripheral lipids defined as virulence factors such as TMM and TDM (Brennan and Nikaido, 1995; Belisle et al., 1997; Grzegorzewicz et al., 2012). TMM can also serve as a means to transport MAcs through the *M.tb* cell envelope (Varela et al., 2012), where enzymes from the Ag85 complex called mycolyl transferases covalently link free MAcs to the AG to form the MAc layer as well as transfer an additional MAc to TMM to form TDM (Belisle et al., 1997; Puech et al., 2002).

Multiple anti-TB drugs target MAc biosynthesis to increase permeability of the *M.tb* cell envelope, allowing for other anti-TB drugs to effectively target DNA/RNA/protein synthesis and energy production. Interestingly, most genes involved in MAc synthesis are considered essential, suggesting that the MAc biosynthetic pathway should be further targeted in the design of new anti-TB drugs (Nataraj et al., 2015). It is thought that increased MAcs – and thus, increased hydrophobicity of the *M.tb* cell envelope – may offer additional resistance for DR-*M.tb* strains. However, it is plausible that the MAc structure rather than their abundance may vary in DR-*M.tb* strains. Increases in MAc carbon chain length would not only explain earlier reports in cell envelope thickness of DR-*M.tb* strains (Velayati et al., 2009a, Velayati et al., 2009b, Velayati et al., 2010), but may also contribute to increased hydrophobicity of the DR-*M.tb* cell envelope, thus allowing for greater protection against more hydrophilic molecules including many anti-TB drugs. Because of this, it is critical to elucidate the role(s) that MAcs play in *M.tb* drug resistance and how changes in MAc abundance and/or structure ultimately influences DR-*M.tb* hydrophobicity, transmissibility, and infection outcomes.

#### 4.2.2 Phthiocerol dimycocerosates and phenolic glycolipids

The biosynthetically related PDIMs and PGLs are predominantly nonpolar lipids that contribute to the hydrophobicity of the *M.tb* cell envelope. Both PDIMs and PGLs are considered important *M.tb* complex virulence factors, with PDIMs only being found in pathogenic mycobacterial species (except for *M. gastri*) (Onwueme et al., 2005; Samanta et al., 2017). In this regard, PDIMs are linked to suppression of the early immune response by controlling TNF and IL-6 production, as well as being potentially involved in phagosomal maturation arrest (Rousseau et al., 2004; Howard et al., 2018; Garcia-Vilanova et al., 2019). Many studies revealed that PDIM-deficient *M.tb* and *M. bovis* mutants have a high degree of attenuation *in vitro* and *in vivo* (Camacho et al., 1999; Cox et al., 1999; Rousseau et al., 2004; Murry et al., 2009; Kirksey et al., 2011; Day et al., 2014), with PDIM-deficient mutants also showing greater susceptibility to anti-TB drugs when compared to the wild-type or complemented strains (Chavadi et al., 2011). PGLs are also produced by pathogenic mycobacteria such as *M.tb* (some strains), *M. bovis*, *M. leprae*, and *M. marinum*. However, the importance of PGLs in *M.tb* virulence is still debatable. Many *M.tb* strains – including laboratory strains H_37_R_v_ and Erdman, as well as the highly transmissible clinical isolate CDC1551 – harbor a mutation in the *Pks15/1* gene disabling PGL production (Reed et al., 2004; Samanta et al., 2017). Conversely, genetic lineage 2 (termed ‘*W-Beijing*’ lineage) *M.tb* strains have an intact *Pks15/1* gene and thus contain PGLs that may contribute to their hypervirulence phenotype described in animal models (Reed et al., 2004; Samanta et al., 2017).

**Figure 2:**
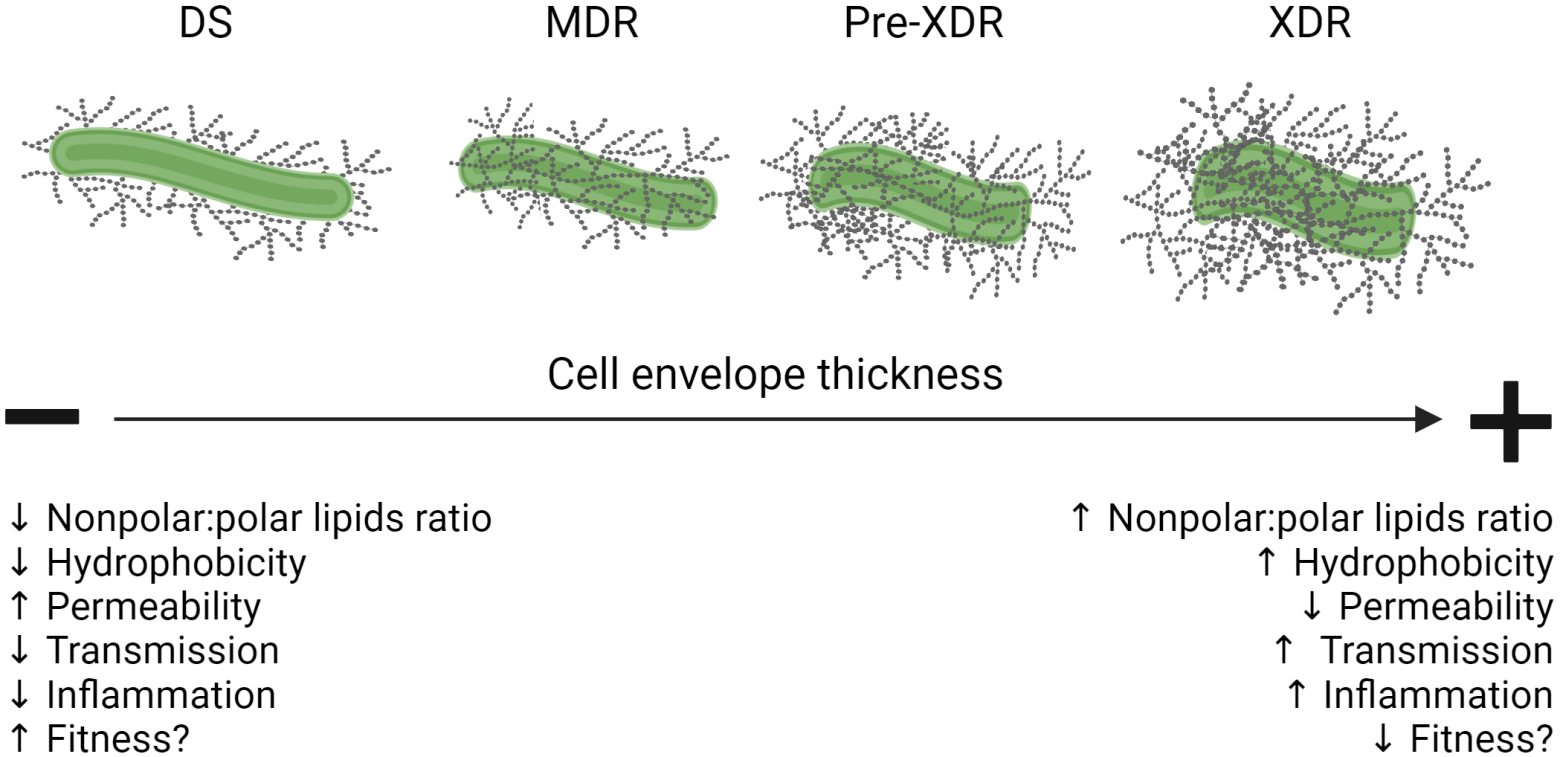
Summary of cell envelope changes in DS- *vs.* DR-*M.tb* and their influence on infection outcomes. Schematic showing that as *M.tb* increases its drug resistance pattern, its cell envelope thickness and hydrophobicity increases, driving reduced permeability and resulting in higher transmission rates while at the same time compromising its fitness within the host by triggering damaging inflammatory responses. Figure created in BioRender. Down black arrows signify a decrease. Up black arrows signify an increase.

Studies have begun examining the relationship between PDIMs on the mycobacterial cell envelope and drug resistance. An initial study using multiple *M.tb* strains showed that the presence of PDIMs is critical for reducing the cell envelope permeability of hydrophobic probes and detergents (Camacho et al., 2001). This study examined *M.tb* strains with deficiencies in genes related to PDIM biosynthesis and transport (*fadD26*, *mmpl7*, and *drrC*) and found that these presented higher permeability to hydrophobic probes, with *M.tb* Δ*fadD26* being the most sensitive to detergents (Camacho et al., 2001; Rens et al., 2021). These results notably did not show any differences in the MIC of multiple hydrophilic anti-TB drugs (INH, PZA, and EMB), indicating that the PDIM-deficient mutants’ susceptibilities did not extend to anti-TB drug effects (Camacho et al., 2001). However, a later study using *M. marinum* Δ*tesA* mutants, a gene encoding a thioesterase that hydrolyses long chain fatty acyl esters involved in PDIMs biosynthesis, showed that PDIM and/or PGL deficiencies are directly associated with hyper-susceptibility to multiple drugs including cefuroxime (CXM), doxycycline (DOX), RIF, ciprofloxacin, and ampicillin (Chavadi et al., 2011). Not only was this finding reproducible in a subsequent study, but it was also shown that another *M. marinum* Δ*papA5* mutant, a gene encoding a polyketide-associated protein with acyl transferase activity involved in PDIMs biosynthesis, had hyper-susceptibility to CXM, DOX, RIF, and erythromycin (Mohandas et al., 2016). Similar findings were shown for both PDIM-deficient *M. bovis* BCG and *M.tb* strains having higher degree of susceptibility to vancomycin (Soetaert et al., 2015). Importantly, RIF-acquired resistance in MDR-*M.tb* strains (particularly through mutation of *rpoB*) is associated with upregulation of the PDIMs biosynthetic pathway (Bisson et al., 2012), increased PDIMs levels (Howard et al., 2018), and changes in over 100 cell envelope lipids (Lahiri et al., 2016). Overall, these data indicate that PDIMs play an important role in the hydrophobicity and permeability of the *M.tb* cell envelope, and that their biosynthesis could be a critical pathway to target when designing novel anti-TB drugs as well as developing a point of care diagnostic tool to distinguish DS*- vs.* DR-TB (**Figure 2**).

#### 4.2.3 Trehalose-containing glycolipids

*M.tb* uses trehalose as both a carbon source and a core component of several cell surface glycolipids known as virulence factors of *M.tb*. The mycolyl-containing trehalose lipids are the most abundant subset composed namely by TDM (6,6’-dimycoloyl-α-D-trehalose) and TMM (6-monomycoloyl-α-D-trehalose). These lipids are comprised of long acyl chains, and it is widely assumed that the mycolates face towards the plasma membrane of the bacterium and trehalose groups face towards the cell envelope surface. Other important trehalose-containing lipids are the acyltrehalose lipids, mainly diacyl- (DAT), triacyl- (TAT), and pentaacyl-trehalose (PAT) (Gautier et al., 1992). These acyltrehalose lipids are valuable components of the *M.tb* cell envelope in that they aid in maintaining the structure of the cell envelope, providing anchors for the outer material layer that thickens the cell envelope (Rousseau et al., 2003; Bailo et al., 2015). Sulfolipids (SL) make up yet another subset of trehalose-containing lipids found on the cell envelope of virulent strains of *M.tb* (Middlebrook et al., 1959; Goren, 1970).

Overall, the role of trehalose-containing lipids in *M.tb* development of drug resistance remains unknown, although trehalose acts as a reactive oxygen species scavenger. Recent studies indicate that trehalose metabolism is associated with both *M.tb* drug tolerance and permanent drug-resistance (Lee et al., 2019). In the presence of INH and RIF, *M.tb* is found to bypass the production of trehalose-containing lipids (Lee et al., 2019). *M.tb* also activates isocitrate lyases involved in the tricarboxylic acid (TCA) cycle to tolerate INH, RIF, and streptomycin after sublethal exposure by circumventing reactive ROS production (Nandakumar et al., 2014). These data suggest that *M.tb* may shift its metabolism (including trehalose metabolism) to circumvent anti-TB drug effects directly linked to the harmful production of ROS (Dwyer et al., 2014; Dong et al., 2015). This is supported by multiple studies indicating that as *M.tb* becomes tolerant to anti-TB drugs in response to a hypoxic environment (e.g. granuloma), it simultaneously downregulates the production of TDM/TMM (Galagan et al., 2013; Eoh et al., 2017). Indeed, *M.tb* drug-tolerant persisters seem to have the capacity to alter trehalose metabolism to survive in a non-replicating state under hypoxic conditions (Eoh et al., 2017). Herein, under anti-TB drug-free environmental pressure, both drug-tolerant persisters and DR-*M.tb* strains can undergo a trehalose-catalytic shift that is absent in DS-*M.tb* strains (Lee et al., 2019). This shift ultimately allows for bacterial survival even when anti-TB drugs cause energy depletion due to the ATP-producing electron transport chain becoming dysregulated (Lee et al., 2019). Alternatively, several studies address targeting trehalose-containing glycolipid transport proteins, such as the mycobacterial membrane protein large 3 (Mmpl3), in the design of new anti-TB drugs (Su et al., 2019; Zhang et al., 2019). Targeting the transport of glycolipids such as TMM to the cell envelope may aid in weakening the cell envelope and possibly increase the efficacy of other anti-TB drugs. Further, the acetylation of *M.tb* proteins plays a critical mechanism of bacterial adaptation to changing environments, being implicated in virulence, drug resistance, regulation of metabolism, and host anti-TB immune responses (Sun et al., 2022; Huang et al., 2023). It is plausible that increased acylation of *M.tb* cell envelope trehalose-containing lipids could be play a similar role (Sun et al., 2022).

#### 4.2.4 Mannose-containing glycolipids and lipoglycans

A predominant family of cell envelope lipids are the mannose-containing glycolipids that include PIMs – glycosylated derivatives of phosphatidyl-*myo*-inositol (PI) – and their associated lipoglycans LM and ManLAM (described in detail elsewhere) (Chatterjee et al., 1992; Chatterjee et al., 1993; Besra and Brennan, 1997; Besra et al., 1997; Chatterjee and Khoo, 1998; Nigou et al., 2003; Torrelles and Schlesinger, 2010; Turner and Torrelles, 2018). Not only are these molecules found in the periphery, but they are also interspersed throughout the plasma membrane and cell wall core (Venisse et al., 1995; Crick et al., 2003; Torrelles and Schlesinger, 2010). Mannose-containing molecules such as arabinomannan, mannan, and manno-glycoproteins are also present on the cell envelope (Torrelles and Schlesinger, 2010), along with mannosyl-β-1-phosphomycoketides (MPM), a mannose phosphate suggested to activate CD1c-restricted T cells (Matsunaga et al., 2004).

The biosynthesis of PIMs is complex, as these are largely heterogeneous molecules that vary in their mannose content, as well as the number and structure of their acyl groups (Garcia-Vilanova et al., 2019). As such, PIMs can be separated into lower- and higher-order structures that coincide with the number of mannose residues (Torrelles et al., 2006). Of the many PIMs found, the most common in the *M.tb* complex cell envelope are PIM_2_ and Ac_1_PIM_2_ (di- and tri-acylated PIM_2_, respectively) as well as PIM_6_ and Ac_1_PIM_6_ (di- and tri-acylated PIM_6_) (Khoo et al., 1995; Garcia-Vilanova et al., 2019). LM is a lipoglycan that is biochemically related to PIMs and thought to be synthesized from PIM_4_ (Gilleron et al., 1999; Guerardel et al., 2002; Guérardel et al., 2003). Along with PIMs, LM is shown to regulate cytokine, oxidant, and T cell responses (Barnes et al., 1992; Chan et al., 2001; Gilleron et al., 2001; Torrelles and Schlesinger, 2010). ManLAM is an abundant mannose-containing macromolecules on the *M.tb* cell envelope and is biochemically related to PIMs and LM (Brennan and Nikaido, 1995; Besra et al., 1997). The heterogeneous structure of ManLAM (described in detail elsewhere) (Chatterjee et al., 1992; Chatterjee et al., 1993; Besra and Brennan, 1997; Besra et al., 1997; Chatterjee and Khoo, 1998; Nigou et al., 2003; Turner and Torrelles, 2018) is defined as containing a mannosyl-phosphatidyl-*myo*-inositol (MPI)-anchor, a carbohydrate core (D-mannan and D-arabinan), and mannose-capping motifs (Torrelles and Schlesinger, 2010). ManLAM biological properties, including its capacity to interact with the macrophage mannose receptor driving phagosome maturation arrest (Fratti et al., 2003; Kang et al., 2005), are described in detail elsewhere (Turner and Torrelles, 2018). Previous studies showed not only that ManLAM is a virulence factor of *M.tb*, but that it varies in size and structure, with certain *M.tb* clinical isolates having truncated forms of ManLAM that is ultimately associated with decreased phagocytosis and lower association with host cell recognition receptors [e.g. mannose receptor (MR), DC-SIGN in phagocytes] (Torrelles et al., 2004; Torrelles et al., 2008).

Efforts have begun to elucidate what role(s) molecules involved in the biosynthesis and transport of mannose-containing glycolipids may play in driving *M.tb* drug resistance. For ManLAM, the ratio of Ara : Man (considered normal at approximately 1.0) (Khoo et al., 2001) varies by *M.tb* strain, with DR- and/or hypervirulent *M.tb* strains showing a greater amount of variability in LAM size and branching (Khoo et al., 2001; Torrelles et al., 2004; Torrelles et al., 2008; Torrelles et al., 2012). Further, a study examining drug resistance of *M. abscessus* showed that mutation of arabinosyltransferase C (an ortholog of *M.tb embC*) resulted in increased permeability of the cell envelope due to the abolishment of LAM biosynthesis driving hypersensitivity to multiple anti-TB drugs (Wang et al., 2022). In a separate study, treatment of *M. bovis* BCG with INH and EMB resulted in increased surface exposure of LAM, which could be linked to the reduction in MAc abundance by the action of INH, as well as an attempt from the bacteria to maintain cell envelope integrity after exposure to anti-TB drugs (Alsteens et al., 2008). This is supported by the upregulation of genes related to LAM biosynthesis following INH treatment (Yimcharoen et al., 2022). That said, this needs to be carefully considered as for this study, the authors used a MDR *M.tb* strain with a *katG* mutation, which should render it insensitive to INH.

Overall, hydrophilic mannose-containing lipoglycans may have low friction within the *M.tb* cell envelope, increasing its hydrophilicity while at the same time reducing its permeability; thus, the biosynthesis pathways of mannose-containing lipoglycans could be important targets for future anti-TB drugs. Indeed, ManLAM/LM structural changes could have a significant impact on the *M.tb* cell envelope integrity, increasing sensitivity to drugs, as well as faster killing by host cells, opening the avenue for combined drug and host-directed therapies.

## 5 The influence of drug resistance on infection outcomes

Fitness has been defined as a comprehensive measure of the bacteria’s ability to survive, reproduce, and undergo transmission, and could represent virulence as well (Zhan et al., 2020). It is true that many mechanistically related forms of drug resistance are associated with reduced bacterial growth (Gagneux et al., 2006b; Andersson and Hughes, 2010; Bhatter and Mistry, 2013; Zhan et al., 2020). This fact in the TB field is debatable; indeed, studies show that INH-resistant *M.tb* strains with mutations in *katG* have decreased virulence, pathology, and fitness in the mouse model (Nieto et al., 2016). However, other studies report that DR-*M.tb* strains range in virulence and that certain drug resistance conferring mutations are at “*non-cost*” and do not decrease fitness (Ordway et al., 1995). This could be explained by the existence of secondary mutations compensating for fitness cost. These secondary mutations could be an indirect, subsequent result of the primary mutations driving drug resistance. For example, an epidemiological study found that 47% of *M.tb* clinical isolates with a *rpoB* mutation conferring RIF resistance also had fitness-compensatory mutations in *rpoA* or *rpoC* (Casali et al., 2014).

Additional factors that can influence infection outcomes include *M.tb* lineage and host genetics and epigenetics that can cause variabilities in the immune response during infection (Saelens et al., 2019; Carey et al., 2022). Previous epidemiological studies suggest that lineage 2 “Beijing” strains are associated with increased virulence, transmissibility, and drug resistance compared to other lineages (Kong et al., 2007; Cowley et al., 2008; de Jong et al., 2008; Thwaites et al., 2008; Niemann et al., 2010; Casali et al., 2014; Carey et al., 2022). Recent reports on DR-TB patients also show cytokine signatures associated with increased TB disease severity and hyper-inflammation, indicating that some DR-*M.tb* strains may have increased virulence (Sampath et al., 2023). However, this conclusion needs to be handled with caution, as it seems possible that DR-*M.tb* appears more virulent in patients simply because it is drug resistant.

Another study assessed the adaptation of *M.tb* and found that strains from multiple lineages have adapted to specific human populations, and that these adaptations are consistent with the geographic spread of each lineage in various parts of the world (Gagneux et al., 2006a). Thus, it is plausible that DR-*M.tb* strains’ primary and secondary mutations, lineage, and host specific genetic and epigenetic factors may account for the vast amount of variability in disease severity and the immune response seen in DR-TB cases. These factors may allow for a small subset of DR-*M.tb* strains with little to no fitness cost to be preferentially transmitted to new hosts, ultimately favoring an increase in DR-TB over time (**Figure 2**) (Zhan et al., 2020).

*M.tb* persisters also complicate our understanding of DR-TB and infection outcomes. These persisters are a subpopulation of *M.tb* that are genotypically the same as the rest of the population, but phenotypically drug tolerant and do not replicate in the presence of drugs (Torrey et al., 2016). Thus, bactericidal drugs do not kill these. Indeed, studies show that clinical isolates with a high-persister phenotype have greater *ex vivo* survival than those with a low-persister phenotype, with specific mutations found in genes related to PDIM biosynthesis in the high persisters (Torrey et al., 2016). However, while persisters can act as a source for drug resistant mutations (Prabowo et al., 2013), the association between drug resistance (not simply drug tolerance) and persister *M.tb* needs to be studied further.

Aside from active TB and latent *M.tb* infection (LTBI), incipient TB (likely to progress to active TB without causing detectable abnormalities) and subclinical TB (does not cause clinical TB-related symptoms, but does cause abnormalities that are detectable using radiologic or microbiologic assays) are also forms of the disease that need to be considered (Drain et al., 2018). In this regard, in 2021 it was estimated that approximately 7 million people were living with subclinical TB, and while these people do not exhibit classic symptoms of active TB disease, they are still able to transmit the infection to others (Kendall et al., 2021). Unfortunately, little is known about incipient and subclinical cases of TB, and even less is known about how drug resistance influences the likelihood of developing these TB stages in comparison to LTBI or active TB. What is clear, however, is that the prevalence of DR-TB is likely to increase if patients presenting subclinical TB are indeed infected with DR-*M.tb* and able to transmit it to others. Thus, understanding how drug resistance influences *M.tb* infection stages and outcomes is an important avenue for further exploration as we work to end TB.

Most studies examining DR-TB are in the context of pulmonary TB. However, there is also evidence of extrapulmonary TB resistance to both first- and second-line anti-TB drugs. Several studies involving extrapulmonary TB indicate that the rate of DR-TB in these patients goes between 4.4% to 20% (Dusthackeer et al., 2015). Similar to extrapulmonary TB, cases of disseminated DR-TB are reported around the world and can also complicate the already difficult-to-manage problem of DR-TB (Jung et al., 2016; Kaplan et al., 2018).

## 6 Concluding remarks

The *M.tb* cell envelope is a unique and complex structure that can adapt to its environment. Key peripheral lipids on the *M.tb* cell envelope contribute to the hydrophobicity of the bacterium and act as virulence factors to influence *M.tb*-host interactions. Although MDR/RR-, pre-XDR-, and XDR-TB have emerged in recent decades, how the cell envelope of DR-*M.tb* changes in comparison to DS-*M.tb* remains poorly understood. Recent studies have started to address genotypic changes naturally occurring in DR-*M.tb* strains that influence the efficacy of anti-TB drugs (Bellerose et al., 2020), and what is currently known about these evolutionary processes and adaptation of DR-*M.tb* are reviewed in detail elsewhere (Allué-Guardia et al., 2021). Examination of other superbugs such as methicillin-resistant *Staphylococcus aureus* (MRSA) showed that MRSA strains can alter their cell envelope to strategically evade the immune response (Gerlach et al., 2018), and that the glycosylation of cell envelope teichoic acids is required for methicillin resistance (Brown et al., 2012). It is possible that we can apply knowledge gained from other superbugs to DR-TB with respect to changes in the cell envelope composition of MDR/RR-, pre-XDR-, and XDR-*M.tb* strains and how these changes impact TB clinical presentation, host responses, and subsequent pathogenesis.

**Figure 3:**
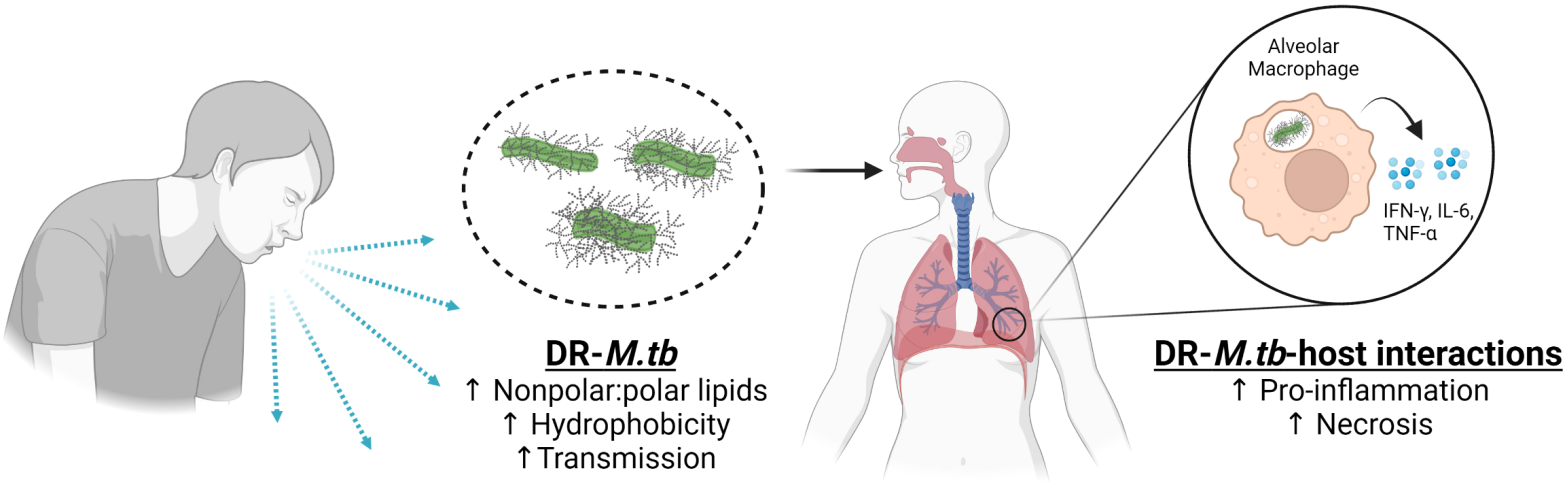
Summary of DR-*M.tb*-host interactions. Schematic showing that DR-*M.tb* strains with increased levels of nonpolar lipids have increased hydrophobicity and transmission leading to the increase in the host cell pro-inflammatory responses in the lung environment, favoring cell death by necrosis rather than apoptosis and driving the progression of the infection. Figure created in BioRender. Up and black arrows signify an increase.

Studies in recent years suggest that the cell envelope of DR-*M.tb* strains thicken, and PDIMs may be playing a determinant role in controlling permeability to hydrophobic drugs. If this is the case, infection with a DR-*M.tb* strain may result in increased virulence, as the host immune system could skew towards a more pro-inflammatory response and increased necrosis of infected cells due to the greater amount of hydrophobic lipids on the cell envelope (**Figure 3**). Another possibility is that, due to the increase in the amount of hydrophobic *vs.* hydrophilic lipids and their associated lipoglycans (*e.g.* PIMs/LM/ManLAM), there is missed opportunity for host cells to recognize hydrophilic lipids that favorably modulate the host immune response towards decreasing inflammation. One outstanding question is whether host-signaling patterns are altered by DR-*M.tb* due to cell envelope changes and, if so, do these alterations influence immune cell recruitment to and granuloma formation at the site of infection? Recent studies have begun to address the genotypic changes in DS- *vs*. MDR-*M.tb*, focusing on mutations that alter the efficacy of anti-TB drugs *in vivo* (Bellerose et al., 2020). However, it will be critical in future studies to also examine pre-XDR- and XDR-*M.tb* strains to assess their natural genetic variations that may influence drug efficacy, if any of these genetic mutations are associated with changes in the *M.tb* cell envelope composition, and how these changes ultimately influence TB pathogenesis.

Another outstanding question related to DR-TB is whether increased amounts of nonpolar lipids (and thus, increased hydrophobicity) on the cell envelope of DR-*M.tb* strains contributes to enhanced aerosolization and transmissibility of DR-*M.tb*, which was previously demonstrated in a study showing that increased hydrophobicity enhances aerosol transmissibility in non-tuberculous mycobacteria (Falkinham, 2003; Jankute et al., 2017). If so, it is plausible that increased hydrophobicity would ultimately allow these strains to be easily transmissible and increase their prevalence over time, thus associating drug resistance with an overall increase in fitness, TB pathogenesis, and impaired host immune responses. Addressing these questions will ultimately aid the TB field in designing novel anti-TB drugs in response to these changes and improving diagnosis of DR-TB in POC settings to reduce transmission and improve public health worldwide.

## Author contributions

AS: Conceptualization, Funding acquisition, Investigation, Writing – original draft, Writing – review & editing. MI: Conceptualization, Writing – review & editing. JB: Conceptualization, Writing – review & editing. JT: Conceptualization, Funding acquisition, Supervision, Writing – original draft, Writing – review & editing.
